# Error, reproducibility and sensitivity: a pipeline for data processing of Agilent oligonucleotide expression arrays

**DOI:** 10.1186/1471-2105-11-344

**Published:** 2010-06-24

**Authors:** Benjamin Chain, Helen Bowen, John Hammond, Wilfried Posch, Jane Rasaiyaah, Jhen Tsang, Mahdad Noursadeghi

**Affiliations:** 1 Division of infection and Immunity, UCL, London, UK; 2 Warwick HRI, University of Warwick, Warwick, UK; 3 Windeyer Building, 46 Cleveland St., UCL,W1F 4JT, London, UK

## Abstract

**Background:**

Expression microarrays are increasingly used to obtain large scale transcriptomic information on a wide range of biological samples. Nevertheless, there is still much debate on the best ways to process data, to design experiments and analyse the output. Furthermore, many of the more sophisticated mathematical approaches to data analysis in the literature remain inaccessible to much of the biological research community. In this study we examine ways of extracting and analysing a large data set obtained using the Agilent long oligonucleotide transcriptomics platform, applied to a set of human macrophage and dendritic cell samples.

**Results:**

We describe and validate a series of data extraction, transformation and normalisation steps which are implemented via a new R function. Analysis of replicate normalised reference data demonstrate that intrarray variability is small (only around 2% of the mean log signal), while interarray variability from replicate array measurements has a standard deviation (SD) of around 0.5 log_2 _units ( 6% of mean). The common practise of working with ratios of Cy5/Cy3 signal offers little further improvement in terms of reducing error. Comparison to expression data obtained using Arabidopsis samples demonstrates that the large number of genes in each sample showing a low level of transcription reflect the real complexity of the cellular transcriptome. Multidimensional scaling is used to show that the processed data identifies an underlying structure which reflect some of the key biological variables which define the data set. This structure is robust, allowing reliable comparison of samples collected over a number of years and collected by a variety of operators.

**Conclusions:**

This study outlines a robust and easily implemented pipeline for extracting, transforming normalising and visualising transcriptomic array data from Agilent expression platform. The analysis is used to obtain quantitative estimates of the SD arising from experimental (non biological) intra- and interarray variability, and for a lower threshold for determining whether an individual gene is expressed. The study provides a reliable basis for further more extensive studies of the systems biology of eukaryotic cells.

## Background

The application of microarray technology to study the expression of thousands of genes in biological samples has become commonplace. The diversity of technologies initially explored has been replaced by a landscape dominated by a small number of proprietary platforms, which differ principally in the type of probe used for hybridisation. Each platform has advantages and disadvantages, and knowledge of technical reproducibility and sources of data variability is crucial to optimising experimental design and data analysis. The reproducibility and comparability of several different platforms has been rigorously examined in the MAQC project, and overall the different platforms give reassuringly similar results, with similar accuracy and sensitivities [1].

We have collected a large number of array data sets from Agilent human genome arrays [2]. The latest releases of these arrays have approximately 44300 features, which include various control oligonucleotides, and a set of 41001 different oligonucleotide 60 mers complimentary to unique human mRNA sequences. Of these 41001, the latest Agilent annotation lists 29,806 as corresponding to known genes and/or ORFs, of which 19392 are unique. Around 12000 probes correspond to as yet unannotated stretches of human genome. The Agilent platform is designed to be used as a two colour system, probing and then detecting hybridisation of two different cDNA samples labelled with different fluorescent dyes on the same array (typically Cy3 in the green channel and Cy5 in the red channel).

Although there are now many published studies using the Agilent arrays, there is a paucity of studies systematically investigating the reproducibility and sources of variation in this microarray platform. There is also relatively little software for implementation of data preprocessing for this platform. Using a set of data generated from this array platform, we dissect the major contributors to experimental variability within the data. We develop a new R package (agilp) to extract data from Agilent raw data files, and implement a robust Loess normalisation to a mean of multiple experiments. This reduces variability to a level which allows accurate and informative comparisons between array data sets collected at different times and by different operators. We also demonstrate that the low level expression detected by the Agilent arrays for the majority of genes in any one cell type likely corresponds to a genuine high degree of transcriptomic complexity, and is unlikely to arise from weak non specific cross-hybridisation. The results of the study thus give confidence to studies which use this technology and quantify important performance parameters which can be used to optimise the design and interpretation of future transcriptomic experiments.

## Results

### Impact of logarithimic transformation

We initially examined a set of 77 data sets ( arrays 1-77 in Additional File 1, Table S1) collected in our laboratory over a period of about three years. In each experiment the Agilent human whole genome expression arrays were probed with a mixture of two cDNA preparations labelled with Cy3 and Cy5 respectively. The Cy3 sample in every experiment was a sample of "reference" cDNA prepared from the Stratagene Universal Human Reference reagent. The same batch of reference RNA was used in all cases, although several different batches of labelled cDNA were made. The Cy5-labelled cDNA was prepared from RNA extracted from monocyte derived macrophages or dendritic cells, or the Mutz-3 leukaemic cell line cultured and/or stimulated in a variety of different ways. A summary of the cell type/experimental stimulus for each array is provided in Additional file 1 Table S1. The Cy5 channel for this first set of arrays therefore always provided data for the experimental sample, while the Cy3 provided data for the reference.

The raw output from the scanner shows a large dynamic range from around 60 to 200000 arbitrary units. These data are commonly log transformed prior to manipulation, in order to make the standard deviation (SD) independent of magnitude, and improve the fit to a Gaussian normal distribution. Both these assumptions were therefore tested on the set of reference arrays.

**Figure 1:**
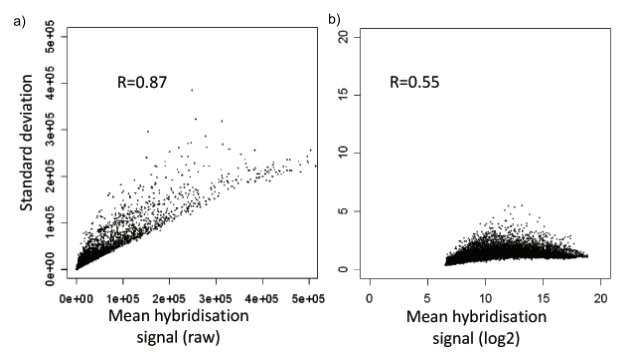
**The relationship between SD and magnitude of signal**. The signal SD calculated for each probe from Cy3 data from 77 reference arrays was plotted against the mean signal. The calculation of mean and SD was done either using raw median signal intensity (left plot) or log_2 _(median signal intensity).

The relationship between SD and mean magnitude of signal across the arrays for each array feature, using raw and log_2 _transformed data is shown in fig 1a. Using a simple linear model, the SD of the raw data can be seen to be magnitude dependent (slope = 0.37, R^2 ^= 0.87). Log_2 _transformation substantially stabilises the SD (slope 0.16, R^2 ^= 0.55)(fig 1b). Further Loess normalisation as described in detail below stabilises the SD even further (slope = 0.06, R^2 ^= 0.22).

The normality of the distribution of the signal across the arrays was tested using the Shapiro Wilk test for each probe individually. The fit varied widely for different probes. However, the proportion of probes whose distribution did not differ significantly from normality (p > 0.05) increased from 55% to 78% when Log_2 _transformed data were analysed. Log_2 _transformed data were therefore used for all further analysis.

### Variation attributable to intra array "experimental error."

Variation in signal between features with the same probe sequence (i.e. probe replicates) provides a good measure of intra-array variation. This variation can be evaluated using 263 oligonucleotides which are replicated 10 times in different positions of the 4 × 4 Human genome Agilent arrays, and a "negative control" oligonucleotide (3 × SLv1) which is present 153 times. The average SD for each set of 10 replicates for each of these 263 probes (including the negative control) was calculated. The calculation was repeated for data from five randomly chosen arrays. The average SD was 0.20 log_2 _units for the Cy5 signal, and 0.16 log2 units for the Cy3 signal (Table 1). The Cy3 and Cy5 signal within each set of replicates within a single array was highly correlated, suggesting the variation was due to local differences (e.g. the efficiency of oligonucleotide synthesis, or the position of the oligonucleotide within the array). However, the SD represented only 2% of the mean log signal for all these replicate probes, highlighting the reproducibility and homogeneity of array construction. Further analysis used the within array average of replicate probes.

**Table 1:** Intra and inter array variation, before and after normalisation

	Within Array^1^	Log transformed without normalisation^2^	Log transformed with linear regression normalisation^2,3^	Log transformed with LOESS linear regression normalisation^2,3^
SD Cy5	0.20	0.91	0.56	0.52
SD Cy3	0.16	0.56	0.31	0.24
Correl Cy5:Cy3	0.84	0.53	0.45	0.40
CoVar Cy5:Cy3	0.02	0.34	0.11	0.09
SD Cy5/Cy3^4^	0.13	0.64	0.43	0.38

**Figure 2:**
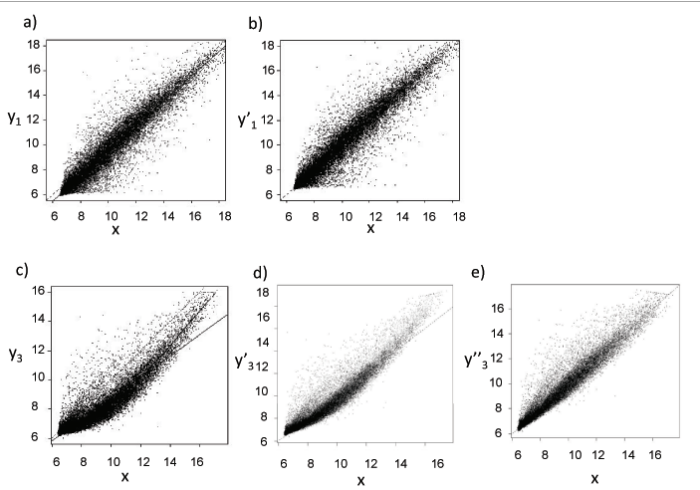
**Data normalisation by linear regression**. y_n _is the Cy3 signal from each probe from array n; x is the mean Cy3 signal from each probe across all 80 arrays. is the linear corrected Cy3 signal from each probe from array n given by: = Y_n_-((α + βx)- x) where α and β are the intercept and slope of the linear regression of y_n_ on x. is the Loess corrected Cy3 signal from each probe from array n given by: where α and β are the set of intercepts and slopes of the Loess linear regression of y_n _on x, dividing the range of x into 10 equal segments. Panels a) and b) show data for n = 1. c), d) and e) show data for n = 3. The dashed line shows x = y; the solid line shows the fitted linear regression. The thick solid line shows the fitted LOESS regression.

1. The SD for Cy3 and Cy5, and the correlation and covariance between Cy3 and Cy5 signals, was calculated across all replicates for each probe which is represented by multiple within array replicates. The Table shows the average of these SDs for all this subset of replicated probes, from a random sample of five arrays.

2. The SD for Cy3 and Cy5, and the correlation and covariance between Cy3 and Cy5 signals, was calculated for each probe across all 77 arrays, and then averaged across all probes.

3. The data were first normalised as detailed in Text and Legend to fig 2

4. The SD of the ratio of Cy5/Cy3 signal was calculated using equation 1 in text.

### Variation attributable to inter array experimental "error."

We next examined the variation which arises from experiments involving different arrays. In order to isolate the variation arising from technical variables (e.g hybridisation conditions, inter array variation etc.) from variation arising from differences in the biological samples being tested, we first analysed variation in the Cy3 signal, which represents hybridisation of an identical biological sample 77 times (Table 1, second column). The average SD for each probe across the arrays for the Cy3 signal was 0.56, representing 6.6% of the mean signal. By comparison, the SD for the Cy5 channel, which includes additional variation from biological variables (e.g. different cell type, different stimuli etc.), as well as unknown intrinsic biological variability was 0.91 (Table 1).

### Reduction in variability by normalisation via linear regression

In order to reduce variability by correcting for systematic differences between arrays we normalised the data by simple linear or LOESS local linear regression against the mean signal of all the samples (i.e. a model in which the mean and the slope is held constant).

To illustrate the effect of the normalisation, the Cy3 probe signal intensities from two sample arrays are plotted against the average probe signal calculated from all the arrays (Fig. 2). It is evident that for the first array, the linear fit is relatively good across the whole range of data, but there is a systematic decrease in signal intensity across the whole range relative to the mean (Fig.2a; R^2 ^= 0.93). The normalisation of this array by subtraction of the regression model provided appropriate correction of the signal values (Fig.2b). In the second example, the linear fit is less good (R^2 ^= 0.857), and a local linear regression model (LOESS ) gives a better overall fit (Fig. 2c). The normalisation of this array by subtraction of the regression model provided appropriate correction of the signal values (Fig.2d,e).

We applied both simple linear and LOESS local linear regression (dividing up the range into 10 local data sets) across the whole set of arrays. Using the normalised data, we calculated the average SD for each probe (the genewise SD) across the set of arrays (Table 1). Linear regression normalisation decreased the average SD of the Cy3 reference array data set to 0.31. LOESS normalisation decreased the SD further to 0.24 (3% of the mean log intensity for the whole array) . 95% of genes had a SD < 0.46 log2 units. The equivalent SD values for the experimental (Cy5) data were also correspondingly decreased (Table 1). We also evaluated quantile normalisation, the preprocessing normalisation implemented in the widely used Affymetrix RMA [3,4] method, which reduced the average SD for the Cy3 channel to 0.29.

### Further reduction of variation by normalisation via ratio to the reference sample

We next examined whether using the ratio of experimental to reference signal for each probe introduces significant further benefit, as reflected in a further reduction in variation of the data. The SD of the difference between the two data sets "reference" and "experimental" is given by:(1)

The covariance between experimental and reference signal was therefore calculated for each probe across the set of arrays (Table 1). The average covariance for all probes was then used to calculate the corrected SD of the difference (ratio) between experimental and reference data sets. Using the ratio for each probe slightly decreased the overall SD of the normalised data (Table 1). The "probe specific" normalisation therefore provides an additional small benefit (in terms of reduction in variability) over and above the global normalisation introduced above.

Unexpectedly, for probes showing the biggest stimulation indices (i.e. the greatest response to interferon), the stimulation index in the green channel is positively correlated to the stimulation index observed in the red channel. This effect is only seen at high stimulation indices, and the correlation is lost once the difference in signal is less than 3 log_2 _units.

**Figure 3:**
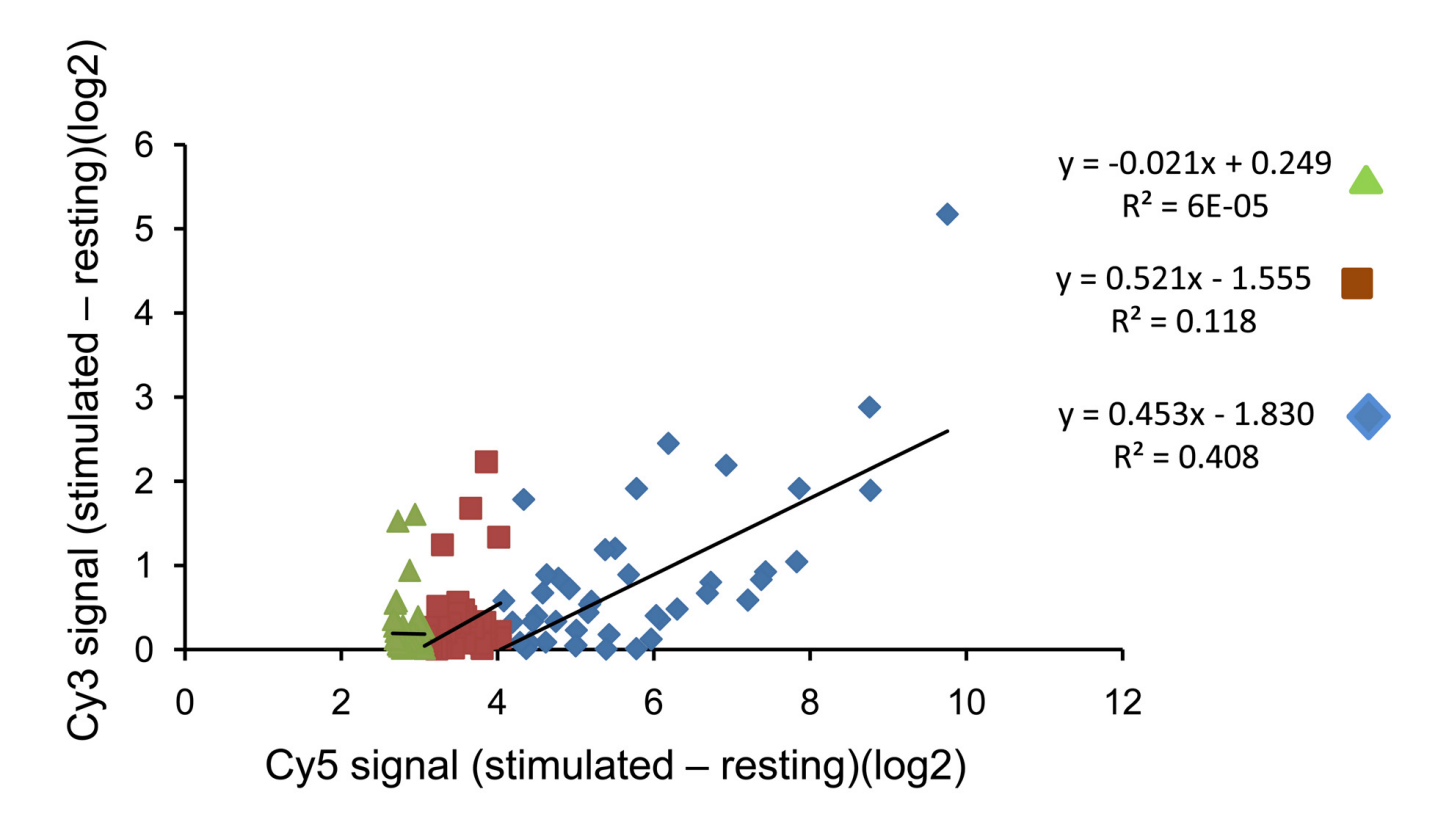
**Interference between Cy3 and Cy5 sample signal**. Loess normalised log_2 _data obtained from a sample of RNA from unstimulated macrophages was subtracted from data from a sample of RNA from macrophages stimulated with IFNβ. The differences in signal in the Cy3 channel are plotted against the equivalent differences in Cy5 channel. The plot shows data for all genes showing a difference (i.e. stimulation index) of >4 log_2 _units (blue), those genes showing a difference of 3-4 log_2 _units (brown) and those genes showing a difference of 2.5-3 log_2 _units. The linear regression line and correlation coefficient for each set of genes was analysed separately.

The phenomenon is illustrated in fig 3. LOESS normalised data was obtained from two sets of arrays of human macrophages, one from a sample of unstimulated macrophages and one from macrophages stimulated for four hours with interferon beta. The signal obtained in the presence of interferon was then subtracted from that in the absence of interferon (i.e. thus deriving the stimulation index) for each gene. The top 50 stimulation indices obtained in this way (i.e. those probes showing the biggest differences between samples) were plotted against the ratios observed for the corresponding green reference channel for the same probe (which should show no "stimulation index", since they come from identical samples) (fig 3). A similar apparent interference (enhancement) of the green signal by a large change in red signal was observed in several other independent experiments. In order to test that the enhancement was not a result of optical crossover (unlikely given the non-overlapping fluorescence spectra of Cy3 and Cy5), an array was hybridised with Cy5 labelled DNA only. The maximum signal observed in the green channel was less than 1% of the equivalent red signal, even at the highest levels of red fluorescence. The molecular basis for the interference between binding of the two differentially labelled samples remains unknown, but the phenomenon has implications both for using red/green ratio measurements, and for experimental design which are discussed further below.

### Low intensity signals in Agilent array hybridisation

**Figure 4:**
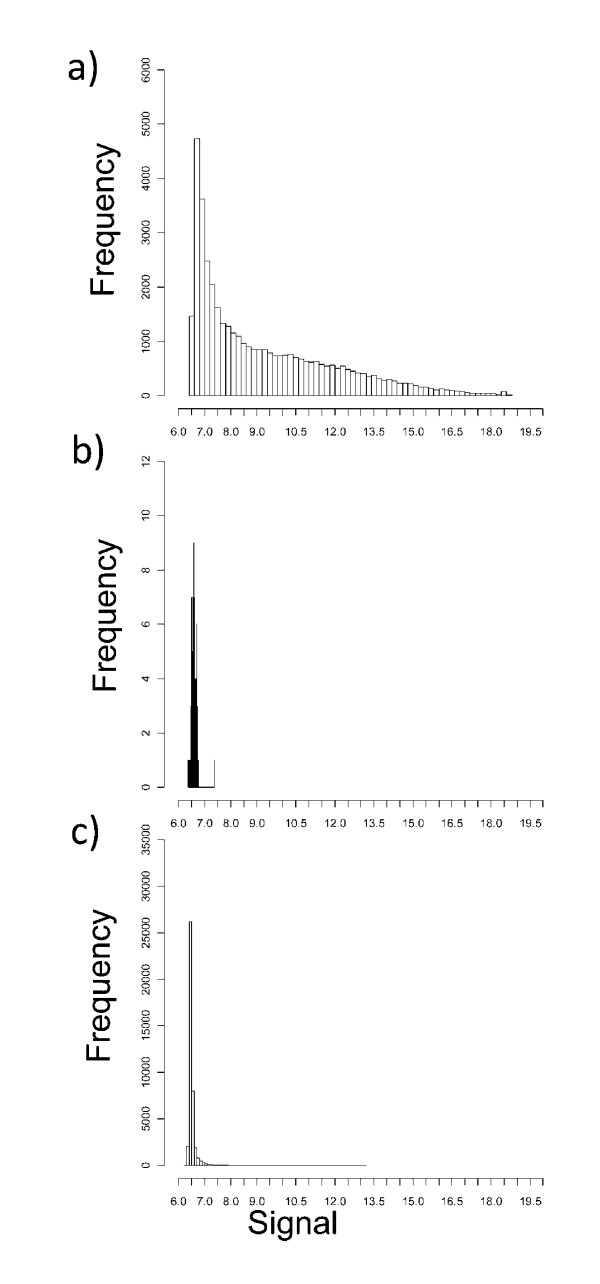
**Log signal frequency distributions**. a) Frequency distribution of the Loess normalised log Cy5 signal of all 41,001 probes from one representative macrophage array. b) Frequency distribution of the Loess normalised log Cy5 signal from the "background" probe (labelled (-)3 × SLv1) for all 96 arrays. c) Frequency distribution of the mean log Cy5 signal for all 41,001 probes from three Arabidopsis samples.

One feature of Agilent arrays is a very large number of probes have a low signal value, close to, but significantly above background. This is illustrated by a comparison between the signal distribution obtained from one representative sample compared to the distribution of the signal from the "negative control" probe provided by Agilent (fig 4a,b).

These data might support a hypothesis that a large number of genes are being transcribed in any one cell type. However, the conclusion is reliant on the the negative control supplied by Agilent. We therefore tested the hypothesis independently. In view of the very large evolutionary distance between Arabidopsis and humans, and the process of Agilent selection of probe sequences so as to avoid repetitive elements or highly conserved common sequences, we predicted that the plant RNA samples should show very little true hybridisation to the arrays. In contrast, if the low level signals were as a result of random low stringency interactions, this should occur to a similar extent using the plant as the human samples. The log signal frequency distribution for the average of three Arabidopsis samples (the data could not be normalised in the same way, since the assumptions that the mean signal is the same is obviously false) was compared to the signal distribution for human samples (fig 4c). The majority of the probes from the arrays hybridised to the Arabidopsis samples gave signals close to or just above the background probe.

**Figure 5:**
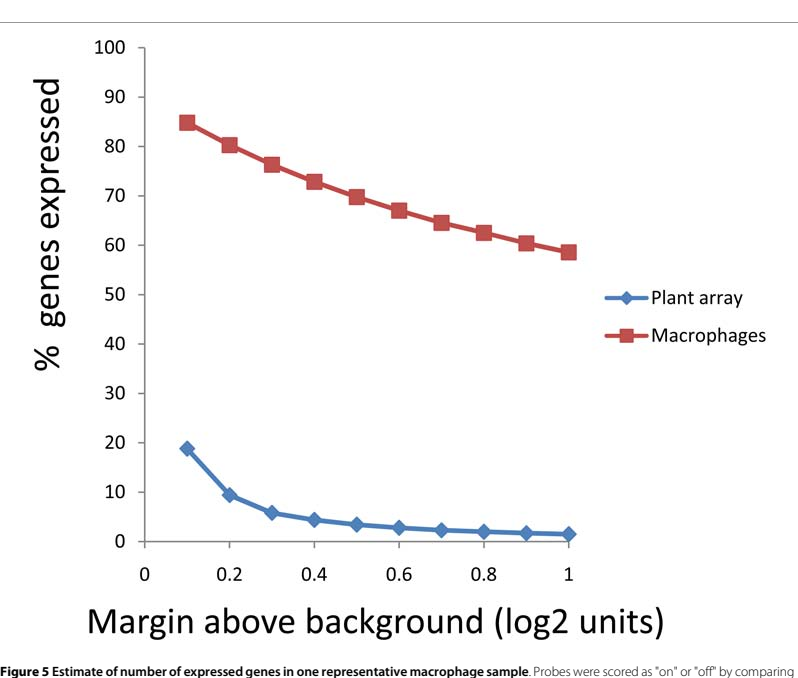
**Estimate of number of expressed genes in one representative macrophage sample**. Probes were scored as "on" or "off" by comparing the signal to the signal from the background probe plus a variable threshold (as plotted along the x-axis). The y axis shows % of probes scored as "on" at a given threshold. Each line shows the average calculated from three unstimulated macrophage samples or three Arabidospis samples as shown.

The data from the Arabidospis samples could be used to calculate an upper limit for "false positive" detection, and hence estimate the proportion of probes showing real positive signals with the human samples (fig 5). In order to analyse the data, we calculated the% of probes showing a positive signal at a range of arbitrary cut-offs above the background probe signal. As discussed above, the % probes showing a positive signal falls off slowly as the cutoff is increased, remaining above 60% up to 0.6 log units above background. In contrast, the % probes showing a positive signal with the plant array samples fell off sharply with cut-off. For example, at a cutoff of 0.5 log units above background, less than 5% probes gave a positive signal, in contrast with 65% when using the human sample.

### Array reproducibility viewed through multidimensional scaling

Having established the basic parameters of error/variability, we examined whether global patterns of gene expression were reproducible and robust when comparing data collected over a time period of several years, and by different operators. The same data set of arrays log_2 _transformed and Loess normalised as detailed above were analysed using classical multidimensional scaling (MDS), a powerful unsupervised approach to identify relationships between sets of arrays. Since the distance matrices are based on Euclidean distance metrics this analysis is mathematically equivalent to Principal Component Analysis.

**Figure 6:**
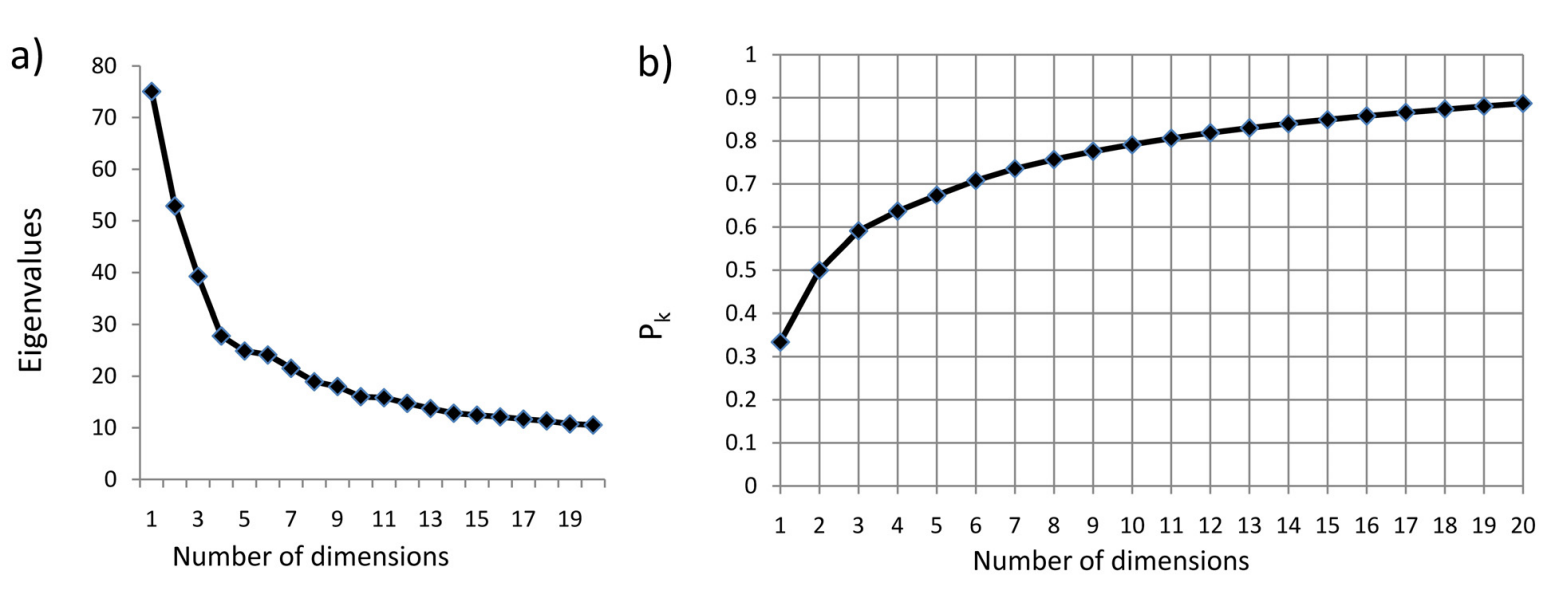
**Chosing the dimensionality of MDS**. The Loess normalised log_2 _data for the Cy5 (experimental) channel for all 77 arrays was analysed as described in the text. a) A plot of the first twenty Eigenvalues as calculated by the mdscale package. b) A plot of goodness of fit value P_k _for 1 - 20 dimensions.

A first step in MDS is to establish the number of dimensions which provides a reasonable compromise between preserving the main features of the data, and minimising complexity. One common approach is to plot the principal eigenvalues (or SDs) to look for an obvious "step" in the slope (fig 6a). There appear to be discontinuities at dimension 4 and again at 10/11. We therefore calculated the goodness of fit parameter P_k _for different numbers of dimensions (fig 6b):(2)

K is the number of dimensions, n is the number of arrays, and λ_i _is the ith eigenvalue. A value of around 0.8 (at which around 80% of the SD has been accounted for) has been proposed as a good cut-off for adequately representing the original data (e.g.[5] ). Ten dimensions were therefore analysed. The justification for including this unusually large number of dimensions is discussed further below.

**Figure 7:**
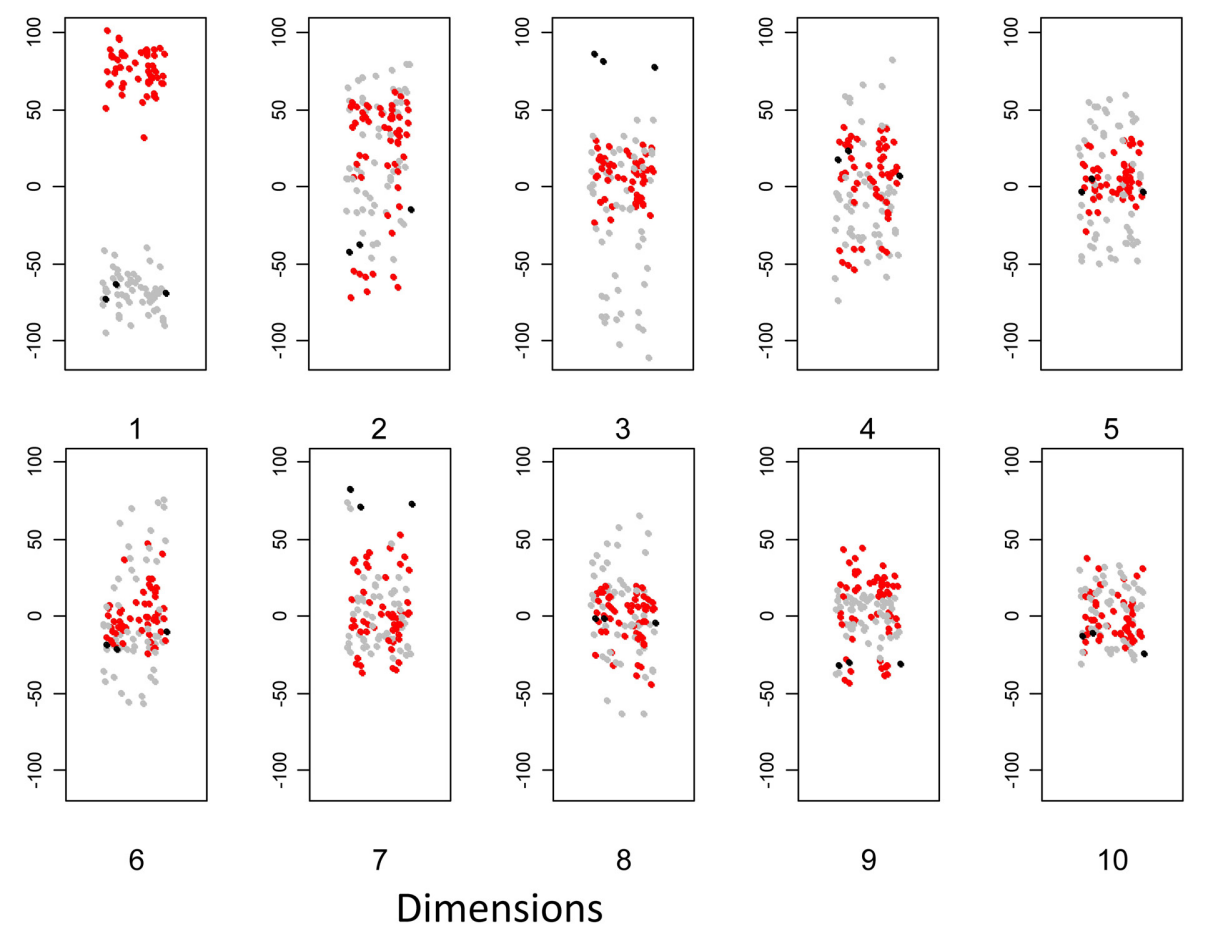
**MDS representation of first set of arrays**. In each plot, the y axis represents the MDS score; a small x axis value is randomly introduced for each point in order to minimise overlap. Each dot represents one array. grey: DC; black: M3DC; red: MDM.

We first examined the distribution of arrays from the point of view of cell type (fig 7). The range of y values decreases with increasing dimension, illustrating the expected decrease in overall variance. The major cell types are clearly segregated within the first few dimensions. Dendritic cells and macrophages are well separated in the first dimension, irrespective of stimulus, or other experimental variable. The dendritic cells derived from the Mutz3 myeloid leukaemic cell line [6] map with dendritic cells in the first dimension but segregate away in dimension 3. The variance in dimension 2, in contrast, seems to reflect differences largely unrelated to cell type.

**Figure 8:**
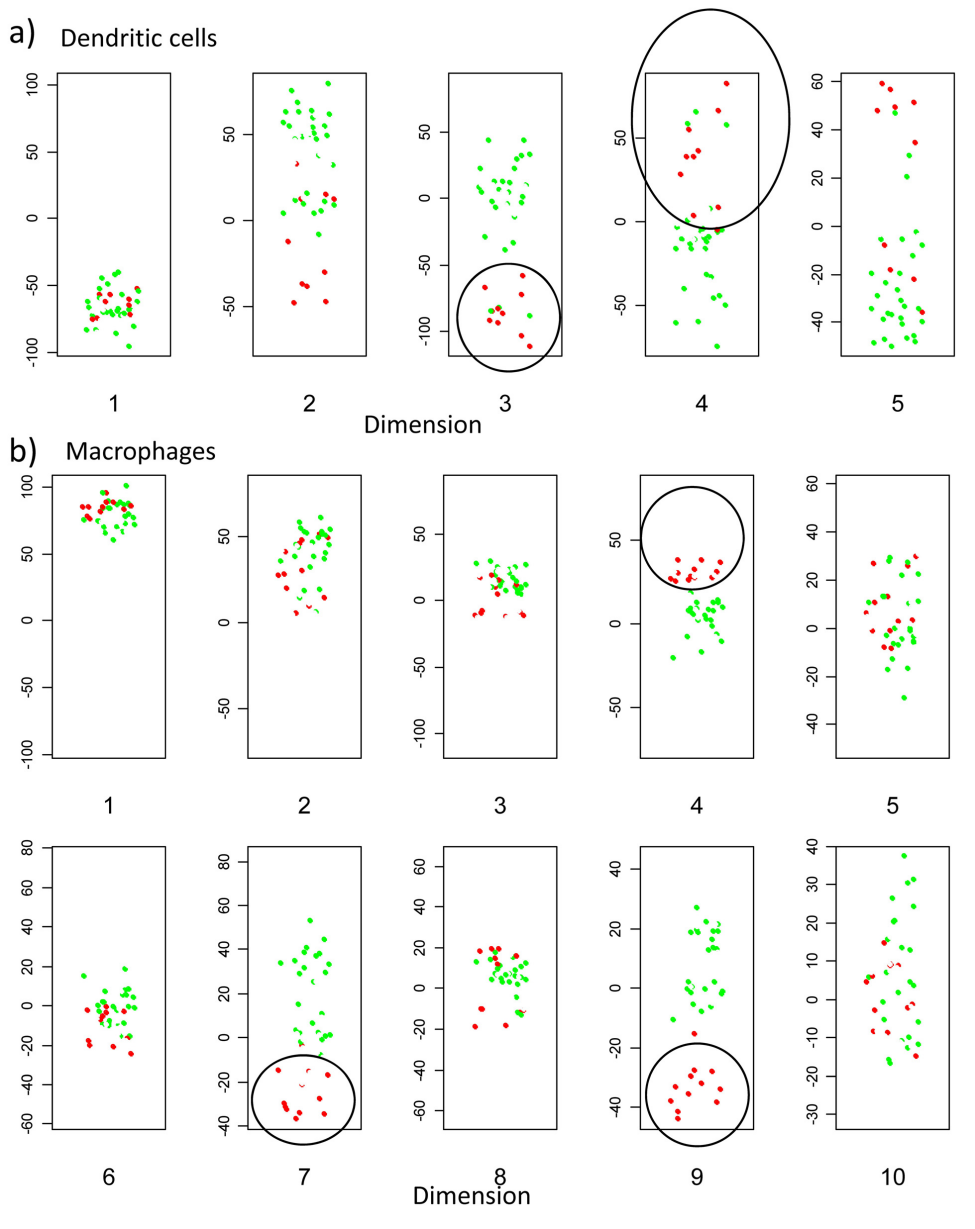
**MDS representation of first set of arrays**. As for fig 7, but coloured according to stimulus. green: unstimulated. red: LPS.

We next examined the distribution in relation to stimulus (fig 8). The dendritic cell response to LPS is clearly identified in dimension 3 and 4. Interestingly, a set of 3 non-stimulated dendritic cell cultures also co-cluster with the lipopolysaccahride (LPS) activated cells. Further inspection revealed that these three cultures were derived from monocytes all from the same individual, suggesting that these dendritic cells may have been already "activated" prior to addition of LPS. Such "spontaneously activated" dendritic cells are a common observation in our experience. A second interesting observation is that the impact of LPS on macrophages (fig 8b) is seen in dimensions different to that for dendritic cells (fig 8a) (i.e. not in dimension 3, but clearly in dimensions 7 and 9 ). These results suggest that the transcriptional response of macrophages and dendritic cells to the same stimulus is made up predominantly of different sets of genes.

**Figure 9:**
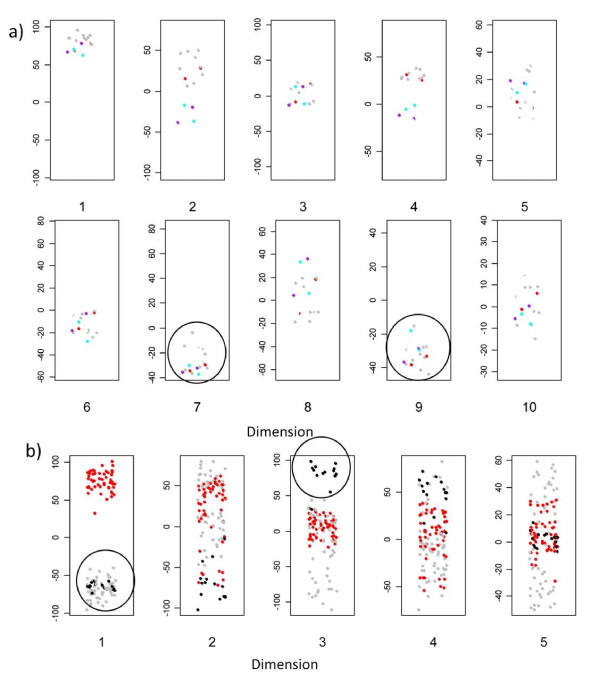
**Analysis of additional samples**. **a) Macrophage replicates**. Two stored Cy5 labelled RNA samples (macrophages stimulated with LPS) were rehybridised with new arrays (purple); the unlabelled original RNA from the samples was also labelled with Cy3, and then hybridised with new arrays (cyan). The dots corresponding to the original hybridisations with these samples are shown in red. The remaining points show all LPS stimulated macrophage samples (grey). **b) Additional Mutz-3 dendritic cell samples (labelled in Cy3 rather than Cy5)**. grey: DC; black: M3DC; red: MDM

We carried out two additional sets of hybridisations to test the reproducibility of the arrays over time. Two old samples of macrophage RNA (LPS stimulated ) were relabelled in Cy3, and hybridised to new arrays together with old stored Cy5 labelled samples . The old and new arrays cocluster, irrespective of dye labelling or long term storage (fig 9a). In particular, all three sets clearly cocluster with the LPS response group within the key "macrophage LPS response " dimensions 7 and 9. Interestingly, the different repeats do segregate away from each other in other dimensions (e.g. 4), suggesting that differences due to experimental protocol can be distinguished from true biologically interesting changes using this multidimensional approach to data analysis.

In addition, we analysed a set of 12 newly prepared Mutz-3 derived dendritic cell arrays (fig 9b) including a set of three additional unstimulated cultures (i.e. biological replicates). All twelve sets cocluster with the old Mutz-3 dendritic cells in dimensions 1 and 3 (i.e. away from monocyte derived DC in dimension 3). Experimental variation which may reflect batch differences between the first and second set of experiments is also detectable in higher dimensions (not shown).

## Discussion

In this study, we have developed a pipeline for processing and analysis of data obtained using the Agilent human 44 K expression array platform. We have assessed commonly adopted preprocessing computational procedures, tested reproducibility of the method and the determinants of variability. Interestingly, investigation of the true background signal intensity, suggested that there is low-level transcriptional activity across most of the genome even in highly differentiated macrophages and dendritic cells. Finally, we have illustrated the application of multidimensional scaling to interrogate complex relationships between individual expression profiles in a large data set.

We confirmed that logarithmic transformation of the raw data facilitates downstream data processing by significantly reducing the dependence of the SD on the signal intensity, and by improving approximation to a normal distribution for many probe signals. Log_2 _transformation did not completely standardise variance vis a vis intensity, and in situations where this becomes critical, additional more complex alternative transformations can easily be incorporated e.g. [7].

A large number of different approaches to data normalisation have been explored in order to improve the reproducibility of array data, particularly with the objective of comparing data generated from different experiments [8-12] . Many of these were designed for the Affymetrix platform (e.g. RMA, dCHIP, GCOS) and are not readily implemented for the Agilent platform . Nevertheless, we compared the Loess normalisation used in this study to the quantile normalisation which is the basis for data preprocessing in RMA and RMA based packages [3,4]. Quantile normalisation and LOESS normalisation reduced the average standard error of the data similarly. However, as discussed in the original implementation of the RMA normalisation algorithm [3], quantile normalisation may "overnormalise" giving identical or very similar values at the extremes of the quantile range. This problem is less serious in dealing with data from the Affymetrix platform, which uses multiple probe sets per gene, and has inter probe variances often greater than the inter array variance. One of the most detailed published comparisons of alternative normalisation approaches did also include a baseline Loess normalisation procedure similar to the one used here [3]. The study found relatively subtle differences between normalisation techniques, but did not report absolute levels of error, rendering comparison difficult.

The use of two colour ratio normalisation, which is possible with the Agilent platform but not Affymetrix, is in fact implemented in the proprietary normalisation supplied by Agilent. The advantage of this normalisation, over global array normalisations, is that it can theoretically correct for probe specific systematic errors. However, as shown in equation 1, ratios decrease variance only when correlation between channels is high, and noise in the "reference" channel is small. Previous studies have in fact suggested that the Agilent normalisation protocol is sub-optimal. In fact, as illustrated in Table 1, the actual decrease in variance observed by introducing ratio normalisation with the two colour array signals is very modest. This modest gain needs to be weighed against disadvantages of the ratio measure. Firstly, ratios behave counter intuitively in terms of biological processes at both extremes of the signal range, adopting increasingly large negative values which tend to infinity as the experimental signal approaches zero. Secondly, we detected an unexpected interaction between independent Cy3 and Cy5 signals at high signal intensities, although the mechanisms remains unclear. Although the effect is only seen for a small number of genes, it may nevertheless prejudice the outcome of experiments which use a reference sample in one channel. Overall, the use of the two colour format to run a reference array for each experimental sample is not supported by our study, a conclusion in line with previous studies [13].

In summary, the preprocessing steps implemented in this study significantly reduce the variation due to experimental, rather than biological variation. This value provides a good estimate of the overall SD of the signal attributable to the various sources of intrinsic "experimental error". Given the Gaussian nature of log signal variation for most probes, differences between experiments of one log_2 _unit should be detectable even with rather low sample sizes. However, future studies using spike-in controls, and including comparisons to q-PCR data will be required to validate the analysis given here, and to determine the true discriminatory power of the data. More precise modelling, using probe specific variances computed from the large set of reference arrays, or more sophisticated Bayesian inference [14]may further improve the quality of the data.

The presence of a very large number of probes showing low level hybridisation prompted us to try and distinguish background from low level gene transcription. Hybridisation of the Arabidopsis samples established a realistic background cut-off to determine the boundary at which genes may be considered to be ON or OFF, and showed transcriptional activity for approximately 70% within human macrophage samples. This degree of transcriptional diversity suggests transcription is going on across the majority of the genome even in a single cell type, and is in accord with recent estimates of cell transcriptional complexity obtained from deep sequencing [15,16], and also with the recent upward revision of estimates of the frequencies of transcriptional start sites within the human genome [17]. This data cannot however address the question of whether the mRNA message are translated or even complete.

In general, the power of genome-wide arrays lies in its ability to define a global transcriptomic response, made up of complex patterns of interacting genes. In order to analyse the extent to which these patterns remained reproducible and robust in the face of experimental error and variation, we used MDS which is equivalent to principal component analysis for continuous Euclidean distance measurements and retains more of the information than conventional hierarchical clustering.

MDS analysis of the macrophage/dendritic cell data set (after Loess normalisation) confirmed that the key underlying structure of the data set is robust, and that sample relationships are conserved even when samples are collected at different times, and by different operators. For example, the distinction between macrophages and dendritic cells, between normal and leukaemic dendritic cells, and between resting and LPS stimulation were all very robust to underlying experimental variation. Of interest, the biologically meaningful variables such as those described above, usually segregated from the "artifactual" variables (such as when the experiments was carried out, or the array format) into a different dimension. Since the MDS axes are orthogonal this suggests that the biological and artifactual variables are often determined by non-overlapping sets of gene probes, and the identification of these genes is the subject of on-going analysis.

## Conclusion

**Figure 10:**
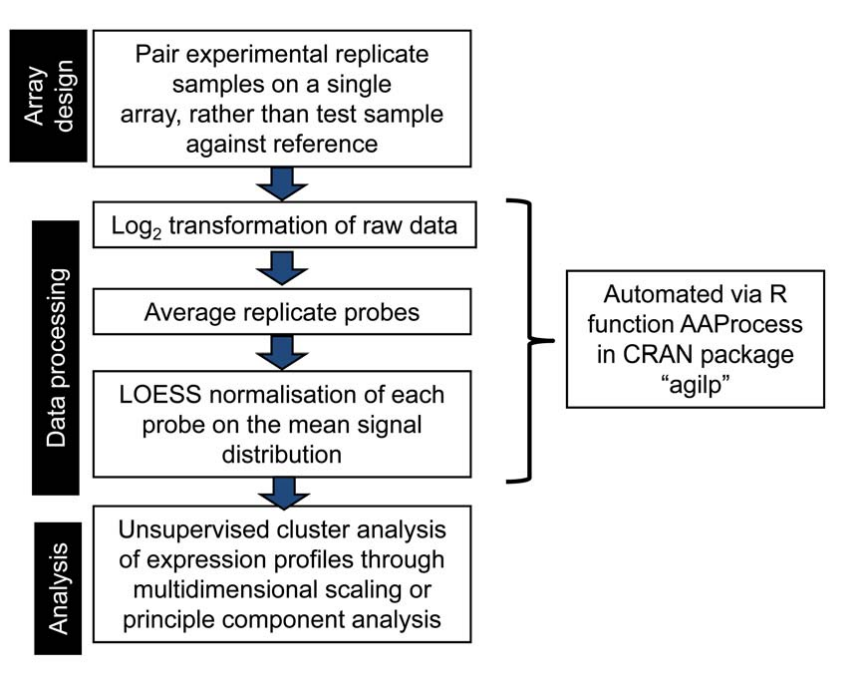
**Pipeline for data collection, processing and analysis as implemented in this study**.

Agilent long oligonucleotide expression arrays represent a robust and increasingly affordable tool for mapping transcriptional activity within the majority of human open reading frames. With correct data processing and normalisation, the genewise variability can be reduced to a few % of the average signal intensity. The pipeline of protocols for data collection, processing and subsequent analysis developed in this study (fig 10) allows robust, and reproducible identification of small gene differences, and reliably preserves the complex transcriptomic relationships associated with important biological variables. Importantly, the common custom of using ratios of signals in the two channels is shown to be redundant, and indeed in certain situations, to reduce the sensitivity of the analysis. Furthermore, the sensitivity of the system allows detection of a large number of low abundance transcripts and confirms the widespread transcriptional activity even within a single cell type.

## Methods

Sample collection. The methods for human cell sample collection, array hybridisation and sample collection have all been described previously [18,19]. A summary of all the samples used is given in Additional File 1 Table S1.

The reference sample is the Stratagene Universal Human Reference reagent, which consists of a mixture of RNA from ten different human cell lines (#740000, Stratagene, Agilent Technologies, Stockport, UK).

Seeds of Arabidopsis thaliana (L.) Heynh. were obtained from the Nottingham Arabidopsis Stock Centre. Plants were grown on 0.8% agar with 0.25 × MS salts, adjusted to pH 5.6 with NaOH, under conditions described previously [20]. At 19 days after sowing, non-senescent, fully-expanded rosette leaves were pooled from 8-22 plants and snap-frozen at -70°C. The experiment was triplicated in three sequential blocks. RNA was initially extracted from tissue samples using a modified TRIzol extraction method [21]. Extracted total RNA was then purified using the 'RNA Cleanup' protocol for RNeasy columns with on-column DNase digestion to remove residual chromosomal DNA (Qiagen, Crawley, West Sussex, UK).

Labelled cRNA samples were generated from RNA samples using the Low RNA Input Fluorescent Linear Amplification Kit according to the manufacturer's instructions (Agilent Technologies, Santa Clara, CA, USA). cRNA was synthesised from the double-stranded cDNA using T7 RNA polymerase, incorporating Cyanine 5-labelled CTP fluorescent dyes (PerkinElemer Life and Analytical Sciences, Boston, MA, USA). Labelled cRNA samples were cleaned using the 'RNA Cleanup' protocol for RNeasy columns (Qiagen) performed at 4°C and eluted using two 30 μL volumes of nuclease-free H2O. Mean dye incorporation for labelled cRNA was 19.53 (± 1.99 SEM, n = 3) pmol dye μg-1 cRNA.

Hybridisation of the Agilent 4 × 44 K whole human genome cDNA microarrays according to manufacturer's instructions http://www.agilent.com. Array images were acquired with Agilent's dual-laser microarray scanner G2565BA (5 μ resolution) and signal data were collected with dedicated Agilent Feature Extraction software (v9.5.1).

The original scanner output files and the normalized microarray data (see below) have been submitted to the ArrayExpress database http://www.ebi.ac.uk/arrayexpress, accession number E-TABM-942). A summary of the samples is given in supplementary Table S1.

The R code for the extraction of raw data from scanner files, removal of duplicates, and Loess normalisation, is available (as function "AAProcess" in package agilp) from the R CRAN repository. http://cran.r-project.org/web/packages/.

The statistical analysis was mostly carried out in R. The functions Shapiro.test and ks.test were used to test for normality. Multidimensional scaling was carried out using the functions distanceMatrix (using Euclidean distance) from the package ClassDiscovery, part of the OOMPA suite of libraries http://bioinformatics.mdanderson.org/Software/OOMPA/, and the function cmdscale from the stats package.

## Authors' contributions

HB and JH provided the Arabidopsis labelled RNA samples, and contributed to revision of the manuscript. JR, JT and WP prepared all the other RNA samples, and carried out all the hybridisations. MN supervised the RNA preparation, labelling and hybridisation. BC carried out the data analysis, wrote the R scripts, and prepared the first draft of the manuscript. The study was carried out under the joint direction of BC and MN. All authors read and approved the final version of the manuscript.

## Supplementary Material

Additional file 1 **Table S1: The details of all the arrays analysed in this study**. An Excel file containing the basic experimental annotation for each array analysed, together with a data base identifier. Click here for file
